# Homogeneous-Heterogeneous Reactions in Peristaltic Flow with Convective Conditions

**DOI:** 10.1371/journal.pone.0113851

**Published:** 2014-12-02

**Authors:** Tasawar Hayat, Anum Tanveer, Humaira Yasmin, Ahmed Alsaedi

**Affiliations:** 1 Department of Mathematics, Quaid-I-Azam University, Islamabad, Pakistan; 2 NAAM Research Group, Department of Mathematics, King Abdulaziz University, Jeddah, Saudi Arabia; Bascom Palmer Eye Institute, University of Miami School of Medicine, United States of America

## Abstract

This article addresses the effects of homogeneous-heterogeneous reactions in peristaltic transport of Carreau fluid in a channel with wall properties. Mathematical modelling and analysis have been carried out in the presence of Hall current. The channel walls satisfy the more realistic convective conditions. The governing partial differential equations along with long wavelength and low Reynolds number considerations are solved. The results of temperature and heat transfer coefficient are analyzed for various parameters of interest.

## Introduction

In the last few decades, the peristaltic motion of non-Newtonian fluids is a topic of major contemporary interest both in engineering and biological applications. To be more specific, such motion occurs in powder technology, fluidization, chyme movement in the gastrointestinal tract, vasomotion of small blood vessels, locomotion of worms, gliding motility of bacteria, passage of urine from kidney to bladder, reproductive tracts, corrosive and sanitary fluids transport, roller, finger and hose pumps and blood pump through heart lung machine. There is no doubt that viscoelasticity has key role mostly in all the aforementioned applications. Viscoelastic materials are non-Newtonian and possess both the viscous and elastic properties. Most of the biological liquids such as blood at low shear rate, chyme, food bolus etc. are viscoelastic in nature. Another aspect which has yet not been properly addressed is the interaction of rheological characteristics of fluids in peristalsis with convective effects. The significance of convective heat exchange with peristalsis cannot be under estimated for instance in translocation of water in tall trees, dynamic of lakes, solar ponds, lubrication and drying technologies, diffusion of nutrients out of blood, oxygenation, hemodialysis and nuclear reactors. The heat and mass transfer effects in such processes have prominent role. The magnetohydrodynamic (MHD) character of fluid especially in physiological and industrial processes seems too much important. Such consideration is useful for blood pumping and magnetic resonance imaging (MRI), cancer therapy, hyperthermia etc. The controlled application of low intensity and frequency pulsating fields modify the cell and tissue behavior. Magnetically susceptible of chyme is satisfied from the heat generated by magnetic field or the ions contained in the chyme. Also the magnetotherapy is an application of magnets to human body which is used for the treatment of diseases. The magnets could heal inflammations, ulceration, several diseases of bowel (intestine) and uterus. With all such motivations in mind, the recent researchers are engaged in the development of model of peristalsis of non-Newtonian liquids through different aspects including heat/mass transfer, MHD etc. Few representative attempts in this direction can be mentioned through the recent researchers [1]–[13] and several studies therein.

To our knowledge, no study has been undertaken yet to discuss the effects of homogeneous-heterogeneous reactions in peristaltic flows of non-Newtonian fluids. Even such study is yet not presented for the viscous fluid. However such consideration is quite important because many chemically reacting systems involve both homogeneous and heterogeneous reactions, with examples occurring in combustion, biochemical systems, catalysis, crops damaging through freezing, cooling towers, fog dispersion, hydrometallurgical processes etc. Hence the main objective of present investigation is to model and analyze the peristalsis of Carreau fluid in a compliant wall channel with convective conditions and homogeneous-heterogeneous reactions. Effects of Hall current and viscous dissipation are also considered. The resulting mathematical systems are solved and examined in the case of long wavelength and small Reynolds number. This article is structured as follows. Section two consists of mathematical modelling and solution expressions up to first order. The behaviors of sundry variables on the temperature, heat transfer coefficient and concentration are discussed graphically in section three. Main results of present study are also included in this section.

## Mathematical Formulation

Consider the peristaltic transport of an incompressible Carreau fluid in two-dimensional compliant wall channel. The channel walls satisfy the convective conditions. The Cartesian coordinates 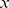 and 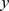 are considered along and transverse to the direction of fluid flow respectively. The flow is generated by the peristaltic wave of speed 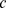 travelling along the channel walls. The Hall effects are also considered in the flow analysis. Further we consider the flow in the presence of a simple homogeneous and heterogeneous reaction model. There are two chemical species 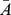 and 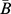 with concentrations 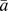 and 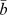 respectively. The physical model of the wall surface can be analyzed by the expression:
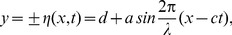
(1)where 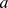 represents the wave amplitude, 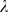
the wavelength, 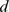 the half width of symmetric channel, 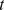 the time, 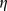 displacement of upper wall and 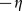 displacement of lower wall.

Consider the uniform magnetic field in the form
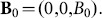
(2)


Application of generalized Ohm's law leads to the following expression
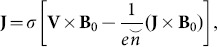
(3)where 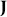 represents the current density, 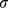 the electrical conductivity, 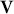 the velocity field, 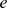 the electric charge and 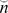 the number density of electrons. Also the effects of electric field are considered absent i.e. 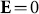


If 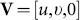 is the velocity with components 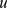 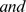 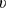 in the 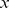 and 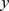 directions respectively then from Eqs. (2) and (3) we have
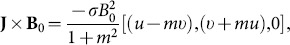
(4)where 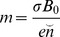 serves as the Hall parameter. The reaction model is considered in the form [14]–[16]:



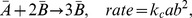
while on the catalyst surface we have the single, isothermal, first order chemical reaction.



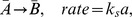
in which 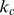 and 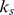 are the rate constants. Both reaction processes are assumed isothermal.

The corresponding flow equations are as follows:
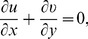
(5)

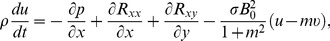
(6)

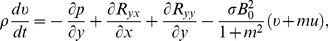
(7)

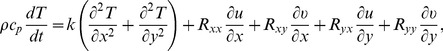
(8)

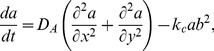
(9)

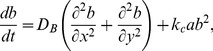
(10)where 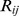 are the components of extra stress tensor for the Carreau fluid and extra stress tensor 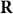 (see refs. [17] and [25]) here is given by



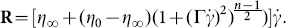
(11)Here 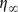 is the infinite shear-rate viscosity, 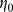 the zero shear-rate viscosity, 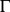 the time constant and 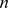 the dimensionless form of power law index 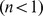. Also 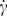 is defined as follows:
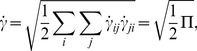
(12)where 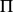 denotes the second invariant strain tensor defined by 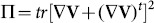 and 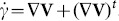 Here we consider the case for which 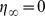 and 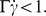 Therefore the extra stress tensor takes the form



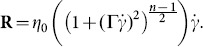
(13)It is worth mentioning that the above model reduces to viscous model for 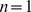 or 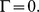 The component forms of extra stress tensor are 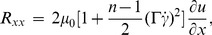
(14)

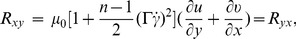
(15)

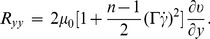
(16)


In above equations 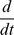 is the material time derivative, 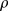 the density of fluid, 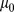 the fluid viscosity, 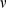 the kinematic viscosity, 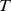 the fluid temperature, 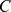 the concentration of fluid, 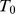 and 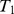 the temperatures at the lower and upper walls respectively, 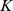 the measure of the strength of homogeneous reaction, 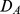 and 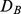 the diffusion coefficients for homogeneous and heterogeneous reactions, 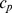 the specific heat at constant pressure, 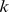 the thermal conductivity, 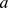 and 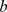 the concentrations of homogeneous and heterogeneous reactions with 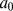 serves as uniform concentration of reactant 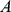 and 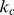 the rate constant.

The exchange of heat at the walls is given by
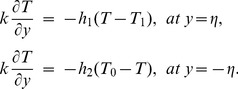
(17)


Here 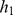 and 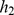 indicate the heat transfer coefficients at the upper and lower walls respectively.

The no-slip condition at the boundary wall is represented by the following expressions
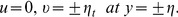
(18)


The compliant wall properties are described through the expression
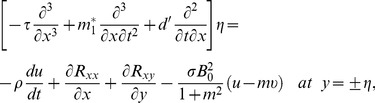
(19)in which 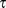 is the elastic tension in the membrane, 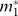 the mass per unit area and 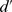 the coefficient of viscous damping. The mass conditions under the homogeneous and heterogeneous reactions are given through the following expressions:



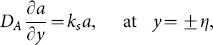
(20)

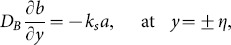
(21)where 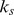 indicates the rate constant.

Performing 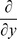 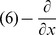 (7) we get
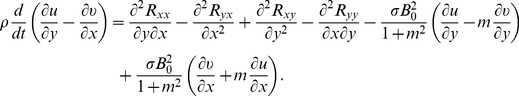
(22)


Introducing the stream function 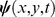 and defining the following dimensionless variables:
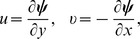


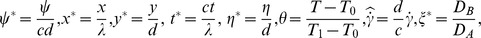


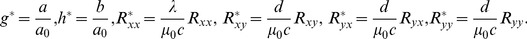
(23)


Eqs. (8), (9) and (22) yield
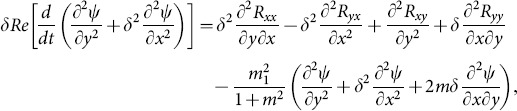
(24)

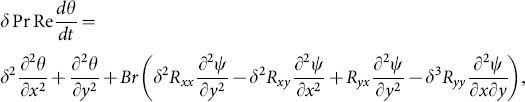
(25)

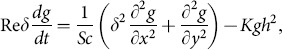
(26)

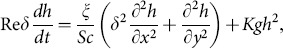
(27)with the dimensionless conditions



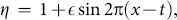
(28)

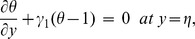


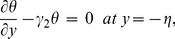
(29)

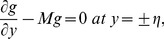
(30)

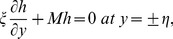
(31)

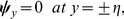
(32)

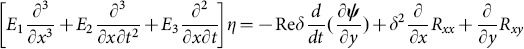


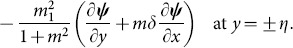
(33)


Also Eqs. (14–16) become
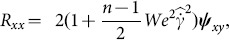
(34)

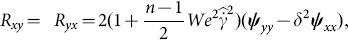
(35)

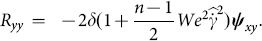
(36)


In above equations asterisks have been omitted for simplicity. Here 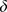 is the dimensionless wave number, the Reynolds number 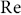, the Prandtl number 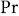, the amplitude ratio 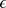, the chemical reaction parameter 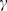, the Hartman number 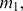 the non-dimensional elasticity parameters 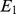, 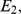 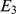, the Schmidt number 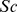, the Eckert number 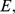 the Brinkman number 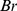, the heat transfer Biot numbers 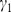, 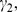 the Weissenberg number 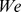, the ratio of diffusion coefficient 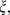 the strength measuring parameters 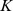 and 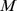 (for homogeneous and heterogeneous reaction respectively) and 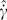 (non-dimensional form of 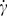) are given through the following variables:
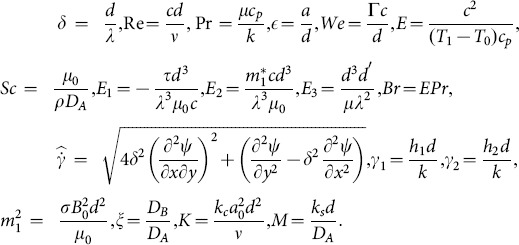
(37)


We now employ the approximations of long wavelength and low Reynolds number [17]–[23] and equality of diffusion coefficients 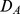 and 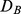 i.e. 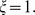 The assumption 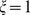 leads to the following relation:
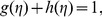
(38)and we obtain the following set of equations



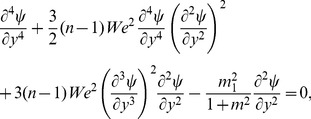
(39)

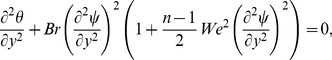
(40)

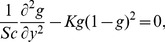
(41)

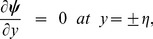
(42)

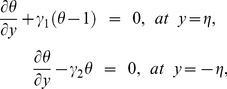
(43)

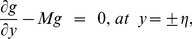
(44)

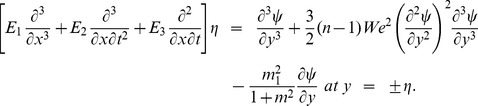
(45)


### 2.1 Method of solution

It is seen from Eqs. (39) and (41) that these Eqs. are non-linear and involve Weissenberg number 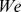 and homogeneous reaction parameter 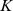 respectively. Therefore the problem at hand cannot be solved exactly, but can be linearized about "small" parameter to the mathematical description of the exactly solvable problem. The technique is referred as perturbation. Perturbation method represent a very powerful tool in modern mathematical physics and, in particular, in fluid dynamics and leads to a series solution of resulting system of equations having small paramter. Therefore we have applied this method to form the series solutions for stream function 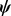, temperature 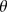 and concentration 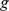 corresponding to the involved non-linear quantities (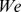 and 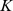). For this we write the flow quantities in the forms:
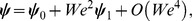


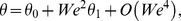


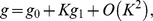


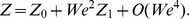



### 2.2 Zeroth order system and its solution

The zeroth order system is given by
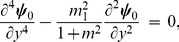
(46)

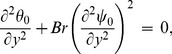
(47)

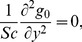
(48)

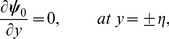
(49)

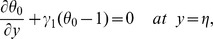


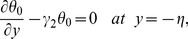
(50)

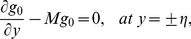
(51)

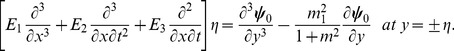
(52)


The solutions of Eqs. (46–48) subject to the boundary conditions (49–52) are
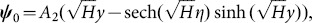
(53)

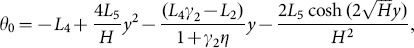
(54)

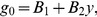
(55)

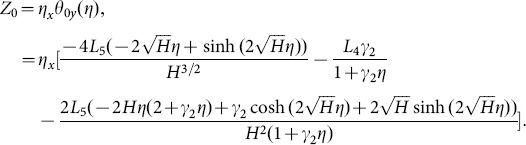
(56)


### 2.3 First order system and its solution

At this order we have
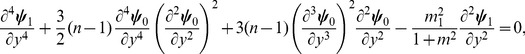
(57)

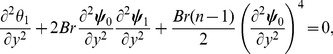
(58)

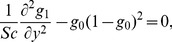
(59)

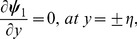
(60)

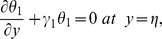


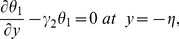
(61)

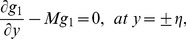
(62)

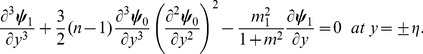
(63)


Solving Eqs. (57–59) and then applying the corresponding boundary conditions we get the solutions in the forms given below:
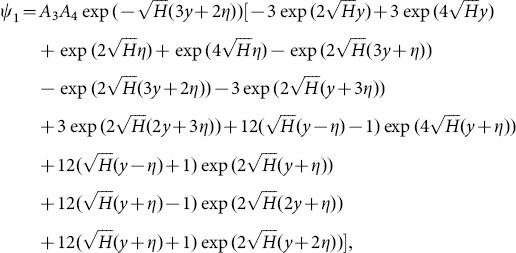
(64)

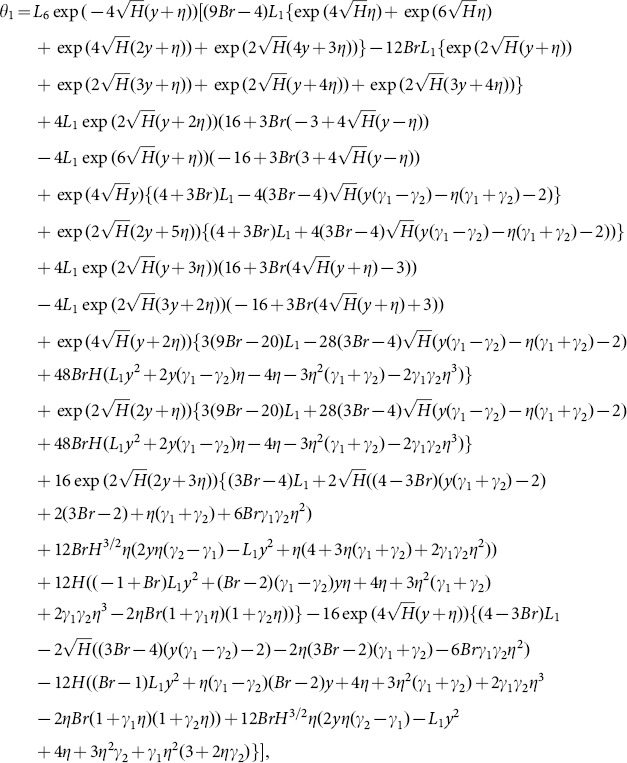
(65)

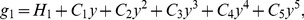
(66)and the heat transfer coefficient is



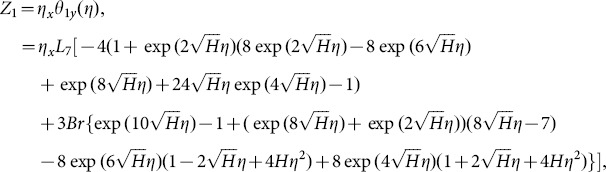
(67)in which
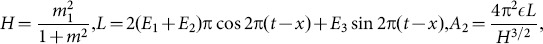


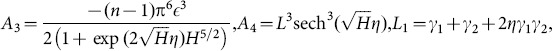


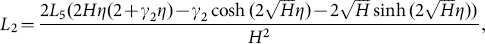





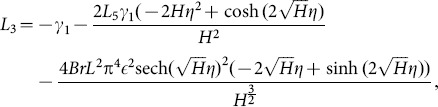


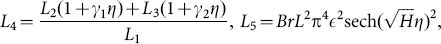





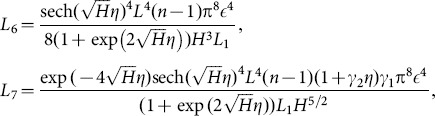


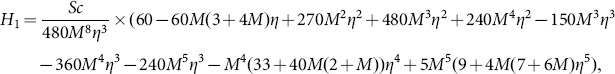


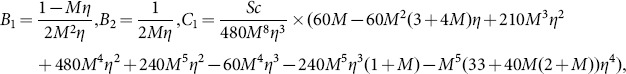


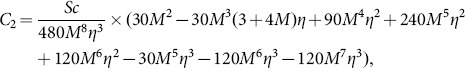


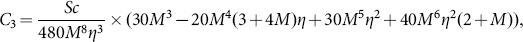


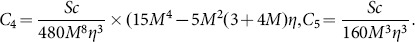



## Results and Discussion

This section is prepared to explore the effects of influential parameters on the temperature, heat transfer coefficient and concentration.

### 3.1 Temperature profile

**Figure 1:**
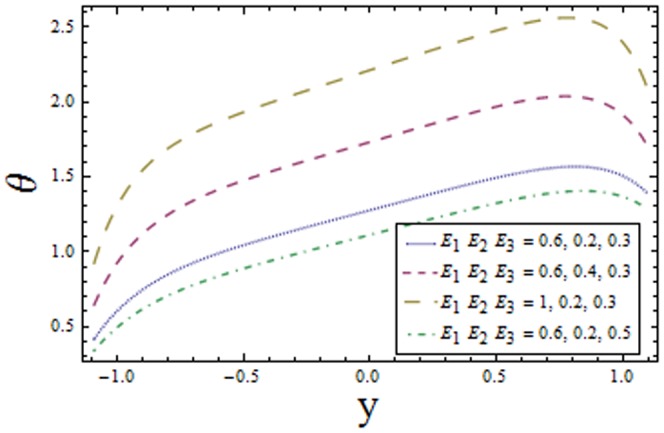
Plot of temperature 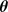 for wall parameters 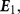 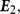 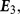 with 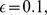 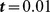, 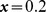, 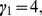 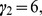 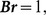 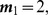 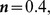 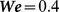 and 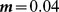

**Figure 2:**
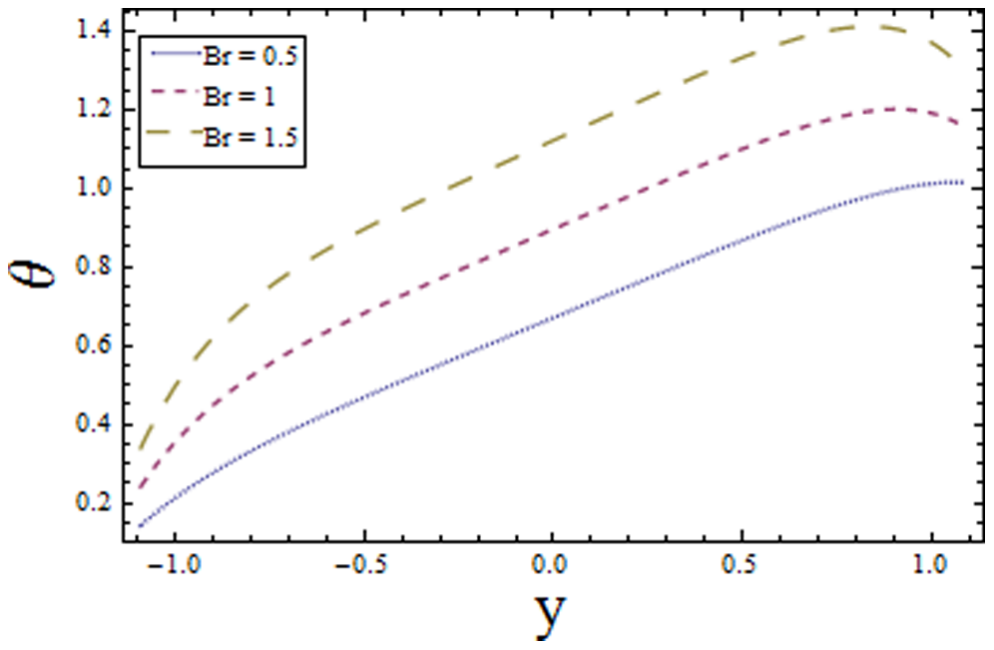
Plot of temperature 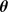 for Brinkman number 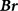 with 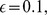 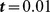, 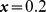, 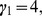 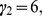 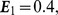 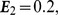 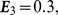 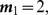 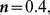 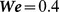 and 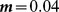

**Figure 3:**
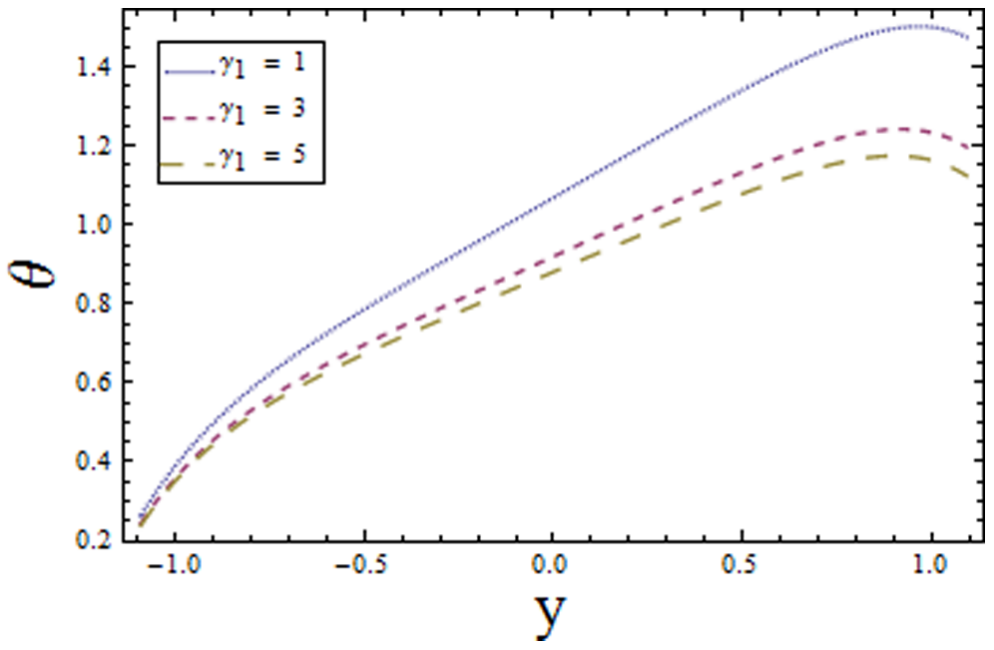
Plot of temperature 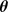 for Biot number 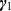 with 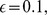 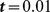, 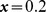, 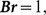 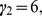 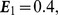 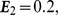 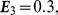 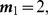 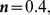 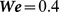 and 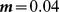

**Figure 4:**
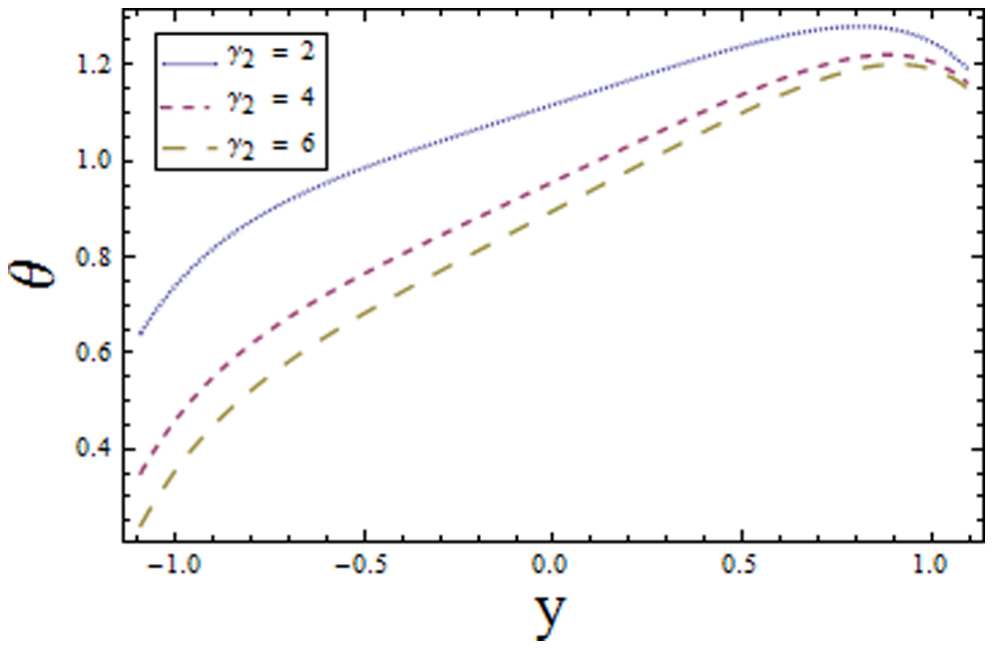
Plot of temperature 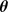 for Biot number 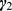 with 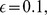 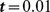, 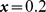, 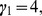 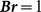 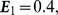 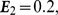 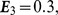 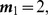 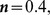 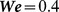 and 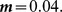

**Figure 5:**
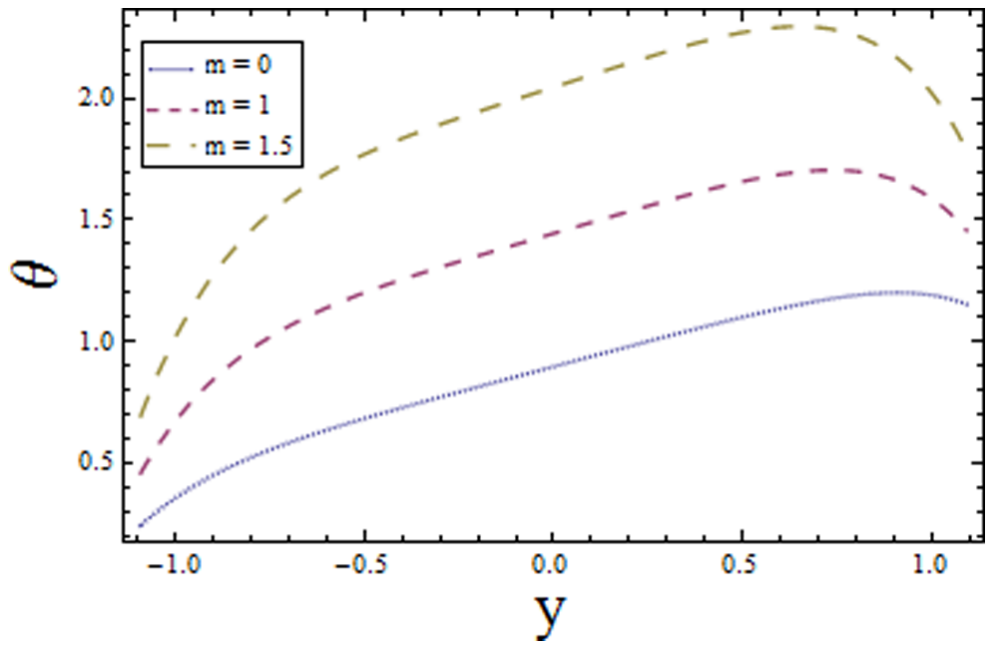
Plot of temperature 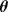 for Hall parameter 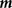 with 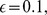 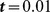, 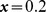, 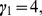 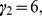 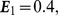 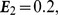 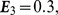 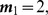 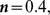 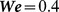 and 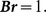

**Figure 6:**
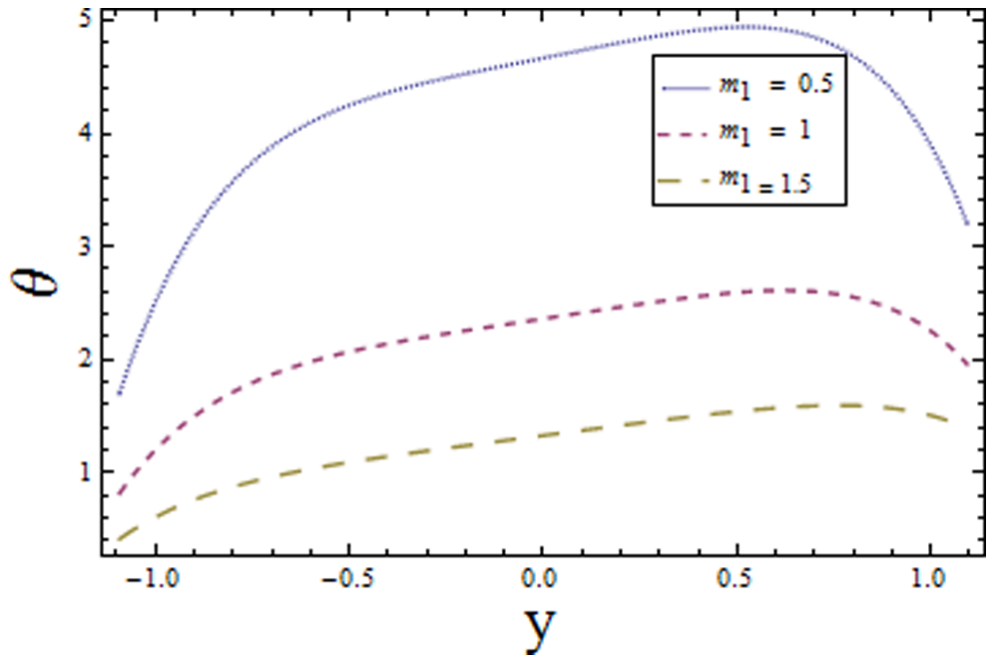
Plot of temperature 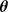 for Hartman number 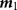 with 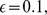 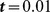, 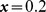, 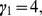 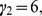 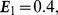 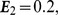 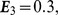 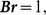 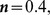 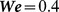 and 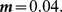

**Figure 7:**
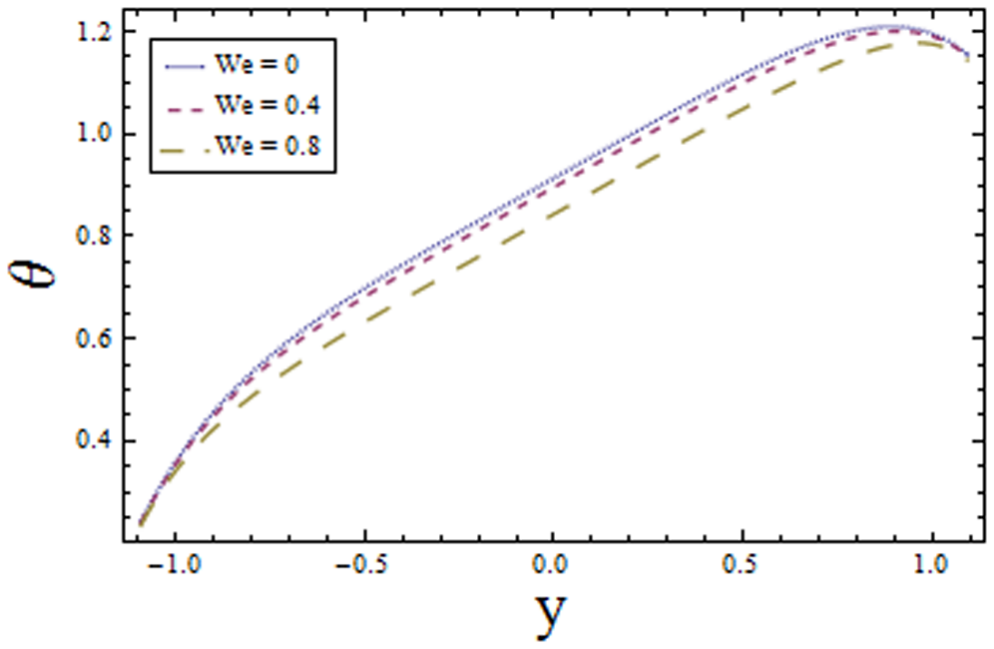
Plot of temperature 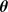 for Weissenberg number 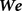 with 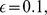 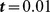, 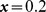, 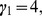 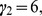 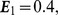 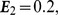 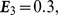 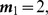 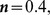 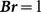 and 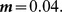

**Figure 8:**
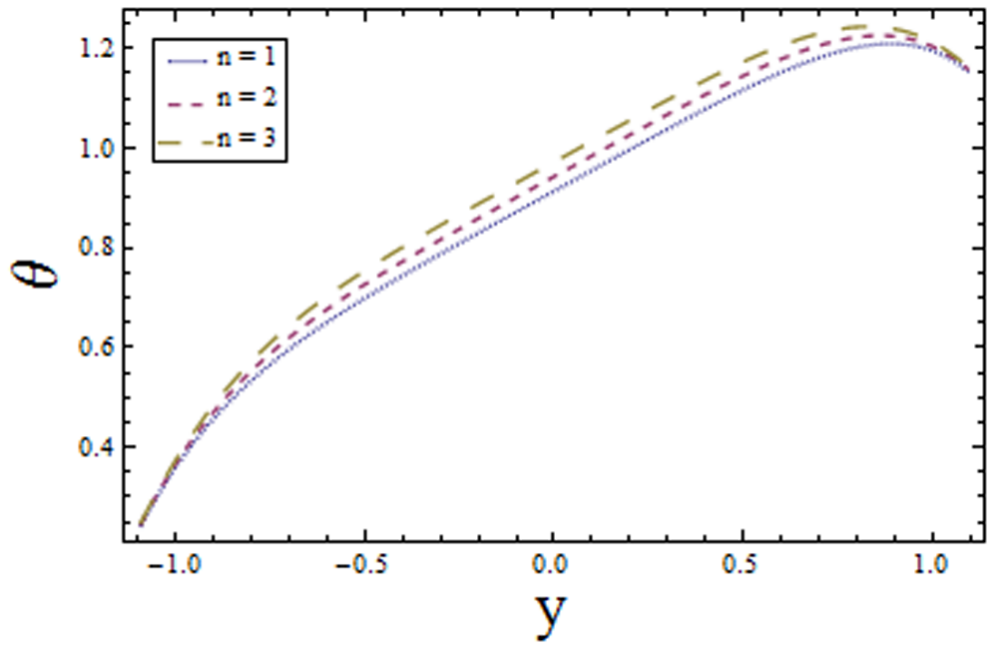
Plot of temperature 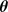 for power law index 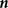 with 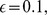 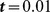, 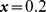, 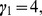 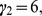 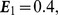 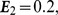 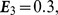 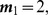 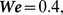 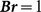 and 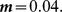

Figs. (1–8) are formulated to examine the impact of various involved parameters on temperature distribution 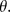 Fig. 1 indicates the increasing behavior of temperature profile with wall parameters 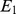 and 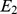 while 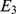 corresponds to reduction in temperature profile. It is in view of the fact that elastic properties of the wall depicted by 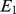 and 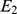 cause less resistance to flow of fluid velocity as well as energy. On the other hand the damping characteristic of the wall identified by 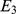 reduces the velocity and temperature of the fluid (see Fig. 1). The temperature profile is an increasing function of Brinkman number 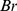 (see Fig. 2). This is because of the increase in internal resistance of fluid particles which increases the fluid temperature. The Biot numbers 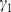 and 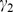 on the lower and upper walls have similar effect on the temperature profile 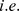 increase in 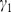 and 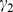 decreases the temperature profile near upper and lower channel walls respectively (see Figs. 3 and 4). It is seen that increasing 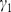 and 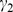 reduces the thermal conductivity which causes reduction of temperature profile. The Hall parameter 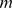 increases the temperature. This is due to the fact that electrical conductivity increases with increasing values of 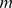 (see Fig. 5). It is observed from Fig. 6 that Hartman number 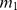 lessens the temperature distribution. Also the results drawn in Figs. 7 and 8 show opposite effects of Weissenberg number 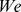 and the power law index 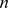 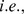 increasing values of 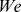 reduces the temperature whereas an increase in 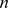 enhances the temperature of fluid. The obtained results are in good agreement with the articles presented in [17]–[19].

### 3.2 Heat transfer coefficient

**Figure 9:**
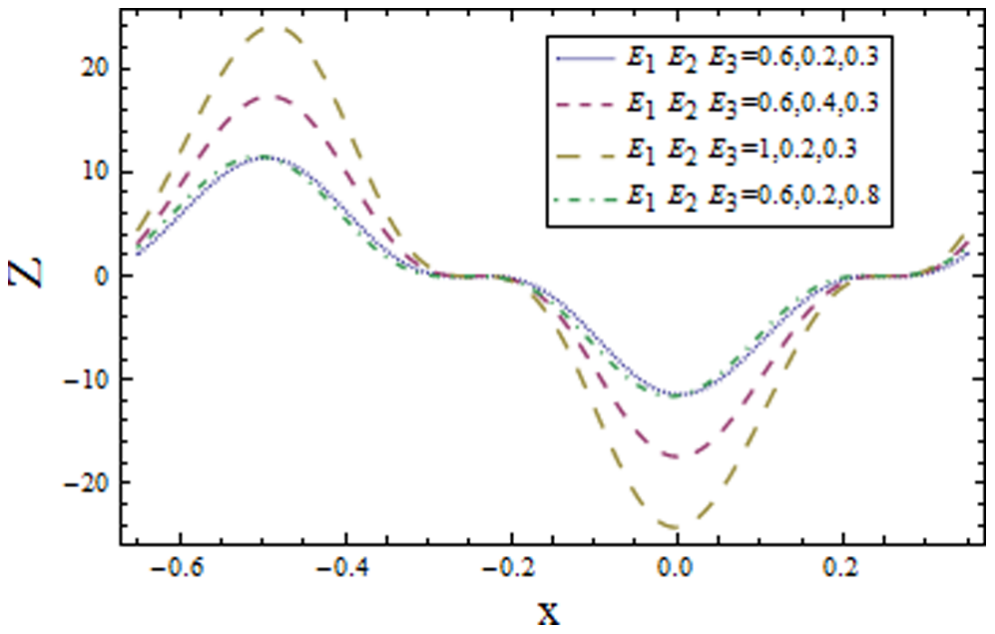
Plot of heat transfer coefficient 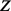 for wall parameters 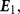 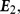 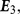 with 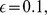 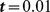, 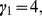 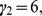 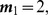 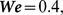 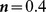, 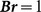 and 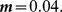

**Figure 10:**
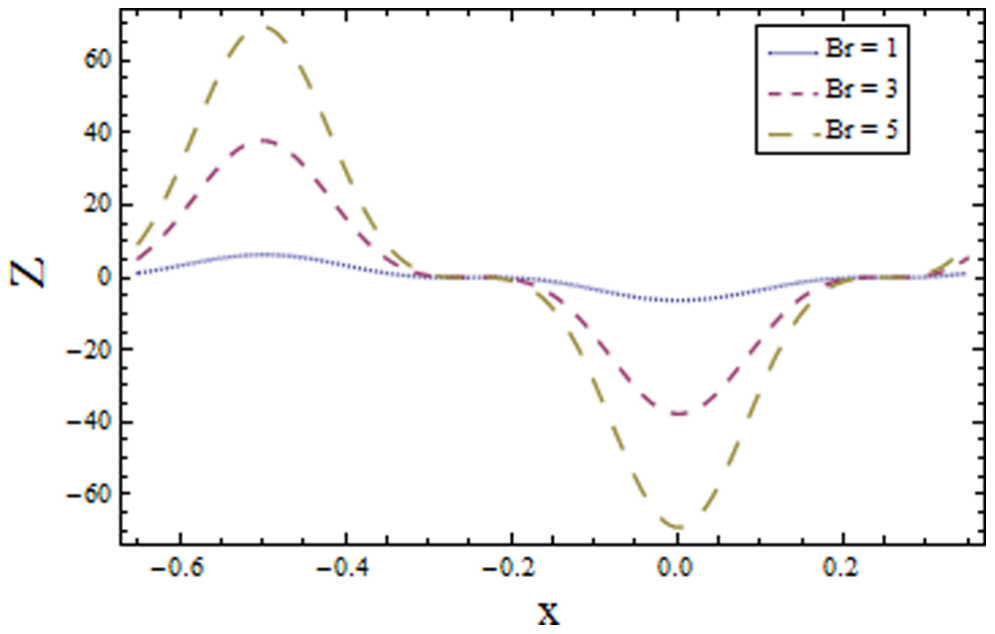
Plot of heat transfer coefficient 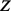 for Brinkman number 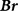 with 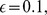 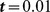, 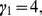 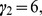 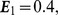 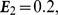 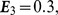 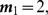 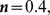 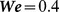 and 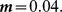

**Figure 11:**
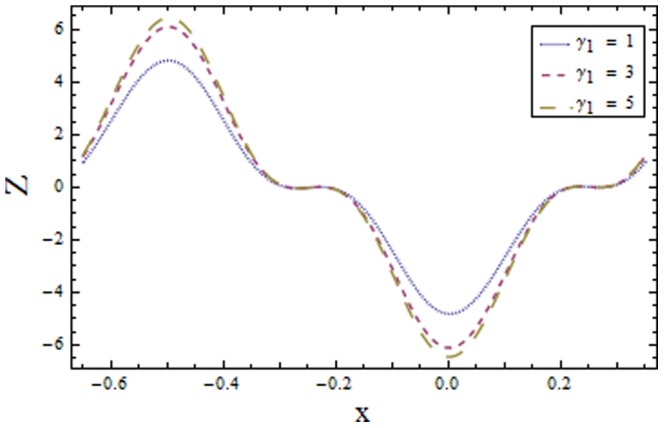
Plot of heat transfer coefficient 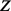 for Biot number 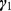 with 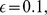 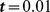, 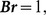 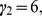 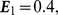 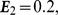 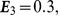 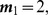 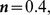 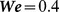 and 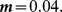

**Figure 12:**
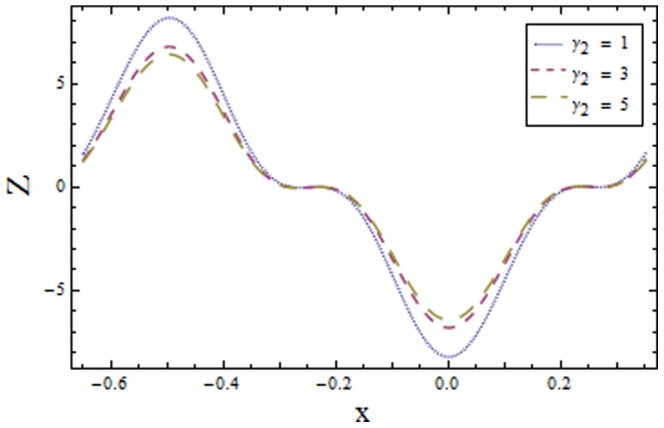
Plot of heat transfer coefficient 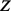 for Biot number 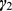 with 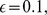 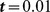, 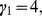 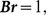 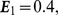 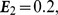 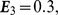 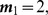 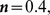 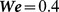 and 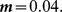

**Figure 13:**
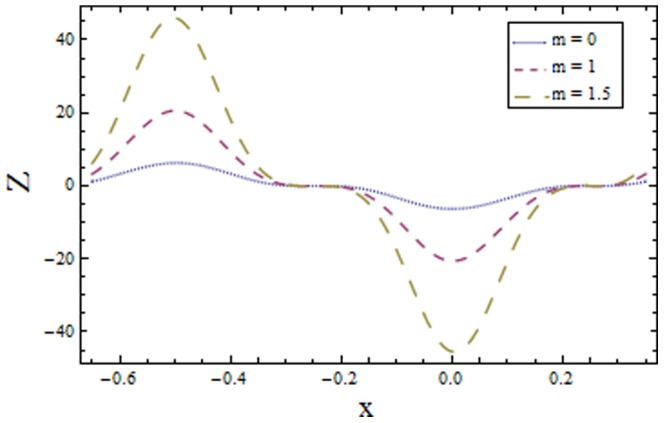
Plot of heat transfer coefficient 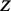 for Hall parameter 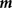 with 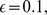 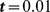, 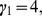 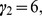 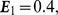 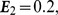 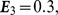 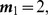 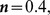 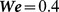 and 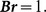

**Figure 14:**
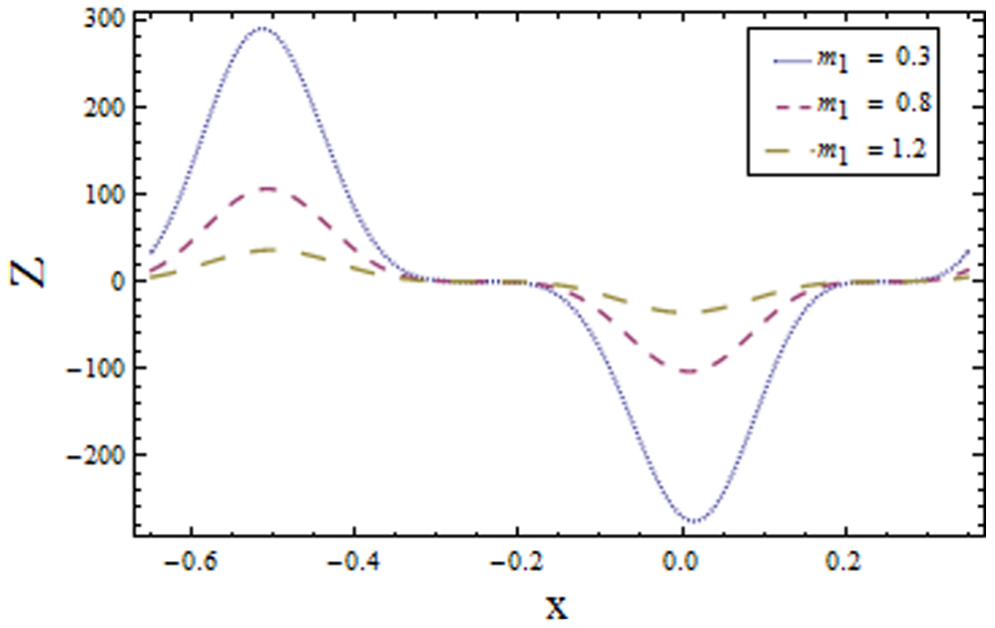
Plot of heat transfer coefficient 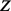 for Hartman number 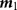 with 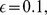 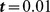, 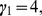 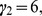 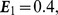 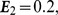 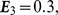 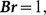 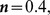 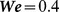 and 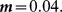

**Figure 15:**
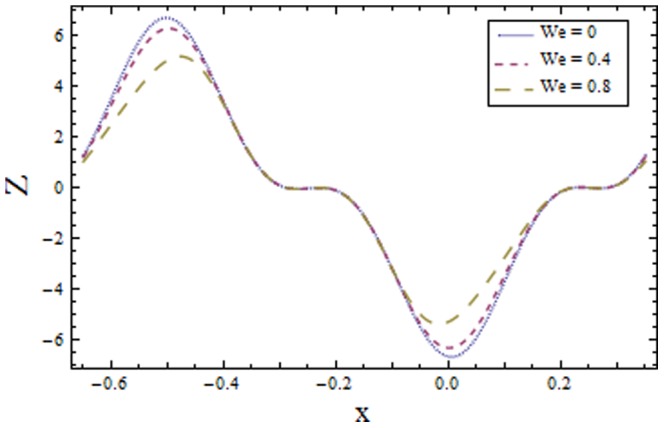
Plot of heat transfer coefficient 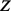 for Weissenberg number 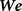 with 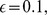 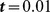, 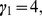 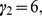 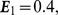 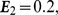 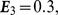 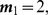 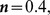 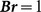 and 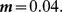

**Figure 16:**
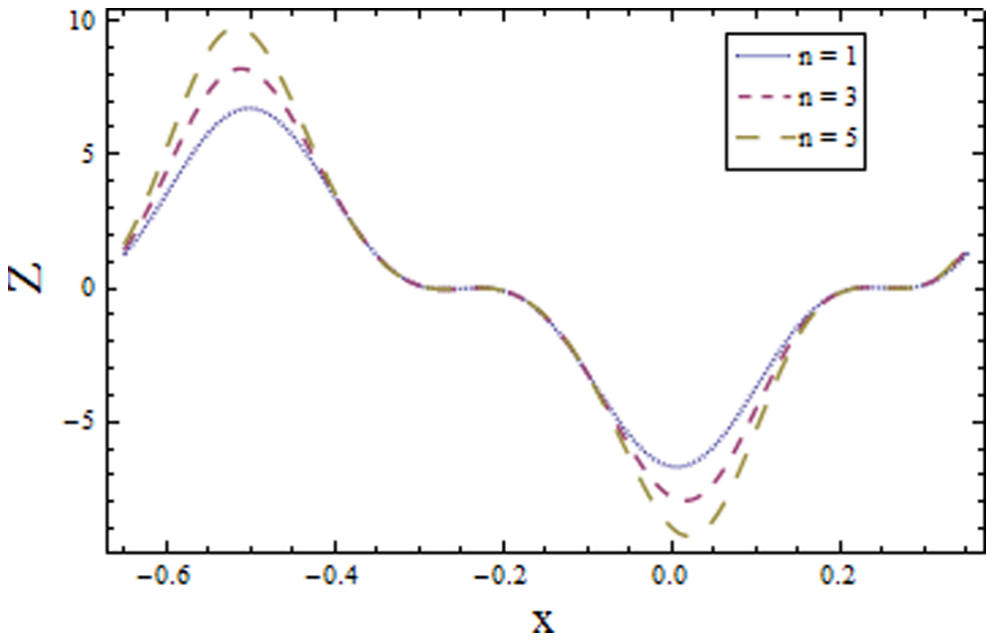
Plot of heat transfer coefficient 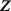 for power law index 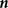 with 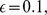 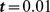, 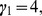 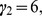 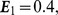 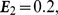 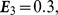 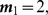 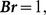 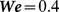 and 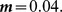

Figs. 9–16 demonstrate the influence of embedded parameters on the heat transfer coefficient 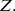 The graphs signify the oscillatory behavior of 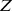 because of the propagation of peristaltic waves. Fig. 9 reveals that magnitude of heat transfer coefficient increases for compliant wall parameters 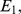 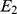 and 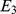. Since 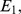 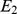 and 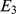 describes the elastic nature of wall that offer less resistance to heat transfer. Increasing values of Brinkman number 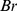 show similar behavior on heat transfer as of wall parameters. However the results obtained are much more distinguished in case of 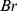 (see Fig. 10). The Biot number 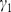 causes reduction in magnitude of heat transfer coefficient on the upper wall. Here thermal conductivity decreases with an increase in 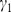 which lessens the impact of heat transfer coefficient near positive side 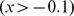 as depicted in Fig. 11. Reverse effect of Biot number 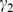 has been observed in the region from Fig. 12 as heat transfer being directly related to Biot number dominates with an increase in 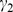 which in turn increases the heat transfer distribution. Fig. 13 shows decrease in heat transfer coefficient 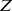 with Hall parameter 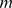. Also in absence of Hall parameter 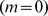 the results are much more distinguished. The Hartman number 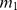 is an increasing function of heat transfer coefficient 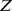 as fluid viscosity decreases with an increase in 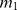. The less viscous fluid particles will move through gain of higher kinetic energy that causes rise in transfer of heat (see Fig. 14). The effects of Weissenberg number 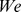 are displayed in Fig. 15. The obtained results show increase in transfer of heat when 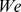 increases as speed of wave increases with an increase in 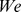 that supports the transfer of heat. The increasing values of power law index show decline in heat transfer distribution (see Fig. 16).

### 3.3 Homogeneous-Heterogeneous reactions effects

**Figure 17:**
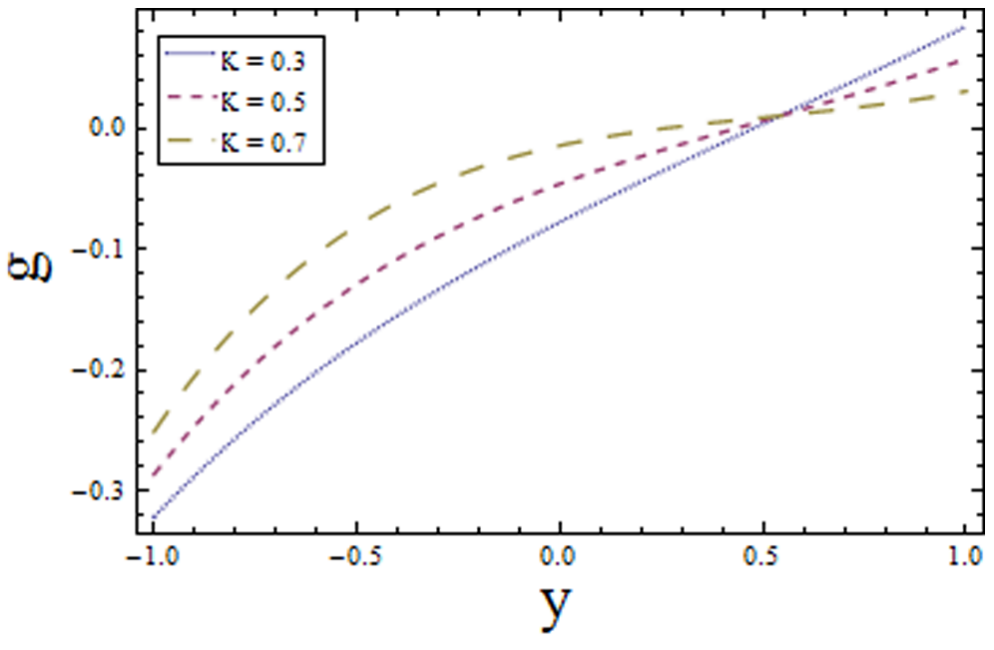
Plot of concentration 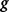 for homogeneous reaction parameter 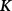 with 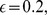 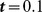, 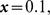 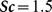 and 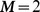.

**Figure 18:**
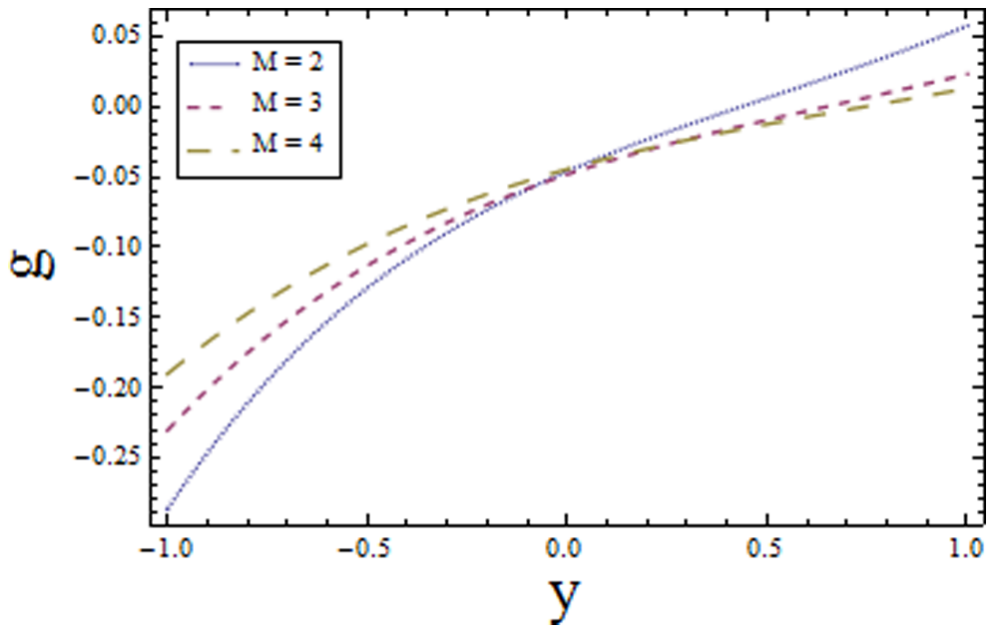
Plot of concentration 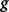 for heterogeneous reaction parameter 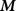 with 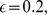 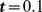, 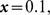 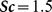 and 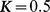.

**Figure 19:**
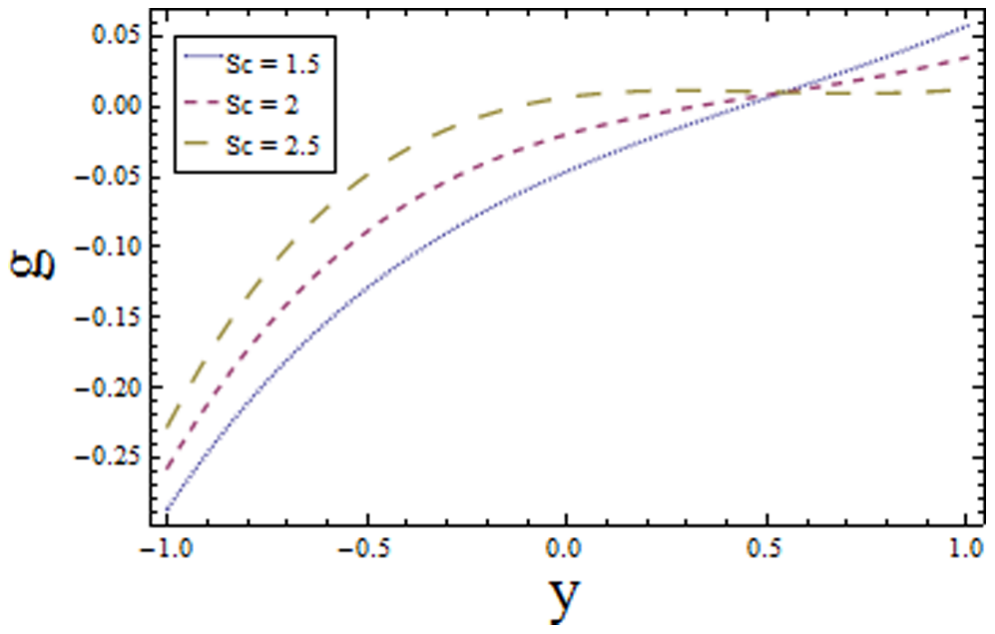
Plot of concentration 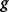 for Schmidt number 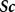 with 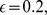 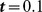, 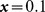 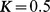 and 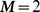.

Effects of homogeneous and heterogeneous reaction parameters 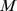 and 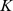 and Schmidt number 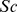 are displayed in the Figs. 17–19. The results drawn in Fig. 17 illustrates the dual behavior of homogeneous reaction parameter 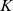 on the concentration profile. It is observed that concentration increases in the region 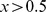 as in this region increase in 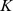 enhances the fluid density hence concentration rises while in the region 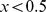 the concentration decreases because viscosity reduces. On the other hand the heterogeneous reaction parameter 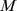 shows the opposite behavior when compared with 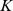 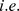 it increases along positive side of the coordinate axes 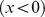 (since diffusion reduces with an increase in 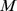 and less diffused particles will rise the concentration) and decreases along negative side of coordinate axes 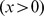 (as increase in rate of reaction dominates the decrease in diffusion in this region). It is evident from Figs. 17 and 18 that the concentration distribution of reactants increases from 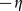 to 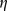 in both cases and after a certain value of 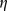 it starts decaying. This critical value of 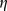 depends on the strength of homogeneous reaction and it is prominent for increasing 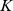. The effects of Schmidt number 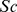 are depicted in Fig. 19. The exhibited results are quite similar to Fig. 17. The drawn results follow by the fact that viscosity of fluid increases with an increase in Schmidt number that provides resistance to flow of fluid. The slow moving fluid particles have small molecular vibrations which lessen the concentration of fluid. As Schmidt number defines the ratio of viscous diffusion rate to molecular diffusion rate. Hence increasing values of 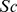 enhances the viscous diffusion rate for fixed molecular diffusion rate which in turn helps to increase the concentration of fluid (see Fig. 19). The similar findings are reported by Shaw et al. [24].

### 3.4 Concluding remarks

The present analysis explores the effects of homogeneous and heterogeneous reactions in the peristalsis of Carreau fluid. Such analysis even for viscous fluid is yet not available. The major results of this study are listed below.

- Similar behavior is observed for compliant wall parameters on temperature profile and heat transfer coefficient.
- Temperature is increasing function of Brinkman number and Hall parameter.
- The Biot numbers and Hartman number decrease the temperature of fluid.
- Opposite effects of Weissenberg number 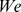 and power law index 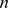 are observed on the temperature profile and heat transfer coefficient.
- Concentration of the reactants is more signified in case of homogeneous reaction parameter 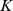 than heterogeneous reaction parameter 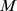.
